# Integrated In Silico, In Vivo, and Deep Learning Approaches in the Discovery of Novel Candidate Molecules for *Aedes aegypti* Control

**DOI:** 10.1002/arch.70138

**Published:** 2026-02-23

**Authors:** Herbert Bezerra Leite, Filipe Alves Ribeiro Rodrigues, Luana Beatriz Rocha Silva, Vanessa Costa Santos, Rosalvo F. Oliveira Neto, Edilson B. Alencar Filho

**Affiliations:** ^1^ Federal University of Vale do São Francisco Petrolina Pernambuco Brazil; ^2^ Department of Computer Engineering Federal University of Vale do São Francisco Juazeiro Bahia Brazil; ^3^ Department of Pharmaceutical Sciences Federal University of Vale do São Francisco Petrolina Pernambuco Brazil

**Keywords:** *Aedes aegypti*, blood meal inhibithors, DeSAO, high throughput virtual screening, juvenile hormone‐binding protein, larvicidal chalcones

## Abstract

The mosquito *Aedes aegypti* is a primary vector responsible for transmitting major arboviruses, including dengue, Zika, chikungunya, and yellow fever. Increasing resistance to conventional synthetic insecticides, combined with their well‐known environmental drawbacks, underscores the urgent need for more selective, sustainable, and effective strategies for vector control. Chalcones have been previously identified by our research group as a promising chemical class of larvicidal agents, with preliminary evidence for distinct mechanisms of action. More recently, an additional strategy for integrated control of *A. aegypti* in its adult stage has emerged through the inhibition of blood feeding, particularly via agonism of neuropeptide Y‐like receptor 7 (NPYLR7). In this context, this multi‐pronged investigation was conceived as a stage‐specific discovery framework addressing distinct biological vulnerabilities of *A. aegypti*. Specifically, the study aimed to: evaluate the larvicidal potential of chalcones through integrated in silico and in vivo approaches targeting juvenile hormone transport; apply deep learning–based high‐throughput virtual screening (HTVS) as a candidate‐prioritization strategy for identifying chemically plausible NPYLR7 agonists associated with blood‐feeding inhibition; and finally generate novel NPYLR7‐oriented molecular scaffolds using DeSAO (“de novo drugs using Simulated Annealing Optimization)” algorithm as a hypothesis‐generating de novo design methodology. These strategies were intentionally pursued as complementary, rather than convergent, discovery axes reflecting the distinct biological requirements of larval and adult mosquito control. Initially, a classical docking‐based virtual screening of 1070 chalcones from the PubChem database was conducted on the *A. aegypti* juvenile hormone‐binding protein (mJHBP), a hemolymph‐circulating protein involved in hormonal regulation of larval and adult development. Docking calculations revealed several analogues with favorable predicted binding energies. Three halogenated chalcones were then commercially acquired for experimental larvicidal assays, which identified 4′‐chloro‐4‐methoxychalcone (2c) as the most active compound after 72 h exposure. In parallel, the Machine Learning driven HTVS and the DeSAO workflow independently identified and prioritized novel molecular scaffolds with predicted NPYLR7 agonist activity, generating chemically plausible candidates for subsequent experimental evaluation of blood‐meal inhibition in adult mosquitoes. Collectively, the results indicate that halogenated chalcones with moderately sized substituents may serve as promising larvicidal candidates, while HTVS and DeSAO provide complementary, chemically diverse architectures for future evaluation in blood‐meal control assays. Taken together, these findings reinforce the value of integrating computational, Machine Learning, and experimental methodologies within a unified pipeline, enabling both validated larvicidal discovery and biologically grounded candidate prioritization for adult mosquito control.

## Summary

In silico and in vivo approaches revealed halogenated chalcones as promising larvicidal agents in *Aedes aegypti;* Deep learning–based Virtual Screening and DeSAO methods identified novel predicted agonists at the NPYLR7 receptor.

## Graphical abstract

An integrated computational/experimental workflow was used to identify new candidate molecules for *Aedes aegypti* control. Docking of 1,070 chalcones against mJHBP, followed by in vivo assays, identified 4′‐chloro‐4‐methoxychalcone (2c) as the most active larvicide candidate. For adult control, a deep‐learning HTVS over one million compounds and a “De novo” design strategy (DeSAO) revealed new NPYLR7 agonistic scaffolds. Together, these approaches provide complementary chemical leads for both larval and adult stages of the mosquito.

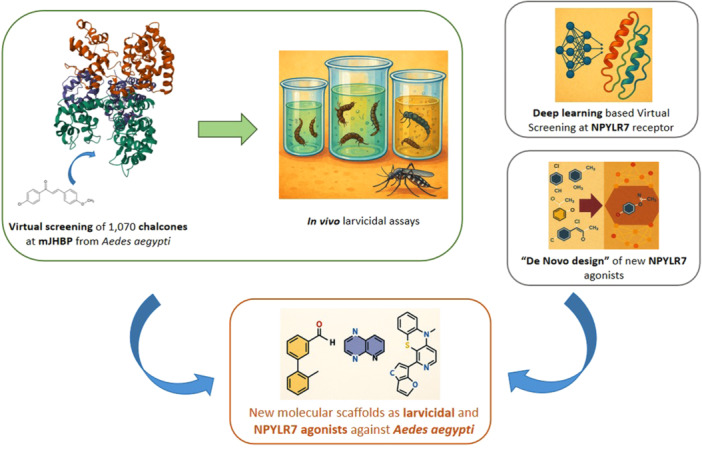

## 1 Introduction

*Aedes aegypti* is a vector responsible for transmitting arboviruses that pose serious global threats, including dengue, zika, chikungunya, and yellow fever (European Center for Disease Prevention and Control 2023). Given the lack of effective treatments for many of these diseases, other than palliative measures, vector control remains an effective strategy (Bangoura et al. 2025). However, the most traditional control method, using chemical products, is limited by the development of insect resistance and the toxic impacts on the environment resulting from the indiscriminate use of synthetic insecticides (Araújo et al. 2023). This reality drives the search for alternative and innovative strategies.

Understanding the physiological mechanisms of target insects is crucial to developing new control agents. Juvenile hormone (JH) is an essential sesquiterpenoid regulator that acts on insect development and reproduction (Hartfelder 2000). During the larval stage, JH is crucial for maintaining the juvenile state by inhibiting metamorphosis until the organism is ready to molt (Kayukawa et al. 2017). In mosquitoes, the mosquito JH‐binding protein (mJHBP), a member of the Odor‐Binding Protein (OBP) family, is responsible for transporting JH, which is highly hydrophobic, into the hemolymph (Kim et al. 2017). Interference with this hormonal pathway induces premature or incomplete metamorphosis, resulting in nonviable larvae or malformed adults, representing an effective vector control strategy.

In this context of identifying protein inhibitors, molecular docking is a computational (in silico) technique that has established itself as a promising tool for the discovery of potential bioactive compounds (Ononamadu et al. 2021). Previous docking studies by our group indicate that the chemical class of chalcones has excellent chemical affinity for two proteins important for the larval stage of *A. aegypti*: hydroxykynurenine transaminase (HKT) and mJHPB itself (Sá 2022; Sá et al. 2023). Chalcones, biosynthetic precursors of flavonoids, are secondary metabolites found in several plant species and exhibit a wide range of biological activities, including antitumor, antiviral, antibacterial, antifungal, and, notably, larvicidal and insecticidal (Sá et al. 2023).

Additionally, traditional docking‐based virtual screening methods face substantial scalability limitations. Even when optimized, algorithms such as AutoDock Vina require several seconds per molecule, meaning that a library of one million compounds would take more than 100 days of uninterrupted CPU computation. This computational bottleneck highlights the need for alternative approaches capable of exploring much larger chemical spaces within practical timeframes. In this context, machine learning–based high‐throughput screening, particularly deep recurrent architectures, becomes not only an innovative strategy but a critical requirement for accelerating the discovery of target‐specific molecules.

Beyond the control by larvicides, adult mosquito management represents a crucial and complementary strategy in the prevention of arboviruses. A recent advance in this field consists of the pharmacological inhibition of blood feeding through the activation of neuropeptide Y‐like receptor 7 (NPYLR7), a G‐protein‐coupled receptor identified in *A. aegypti*. In this context, Duvall et al. (2019) performed an *in vitro* high‐throughput screen (PubChem BioAssay AID 1259423), evaluating 270,665 compounds in a primary cellular assay to identify NPYLR7 agonists. After three successive screening stages, exploratory, confirmatory, and dose–response, the set was progressively reduced from 270,665 to 28 active compounds, of which six selective agonists were functionally validated. These compounds were able to activate NPYLR7 and block host‐seeking and blood‐feeding behavior, both in vitro and in vivo, and mosquitoes with CRISPR‐Cas9 mutations in the NPYLR7 gene were resistant to the action of these agonists, confirming target specificity.

Subsequently, Zeledon et al. (2024) continued this line of research by developing second‐generation agonists based on receptor structural modeling, molecular docking, and structure‐activity relationship (SAR) analyses. The new compounds maintained the quinazoline core as a central scaffold, functionalized with guanidine or amine groups, which conferred high affinity for acidic residues in the receptor binding site. Systematic exploration of these substituents led to the discovery of significantly more potent analogs, some active at nanomolar concentrations, capable of effectively inhibiting biting behavior in adult mosquitoes. These findings solidify NPYLR7 as a promising molecular target for adult mosquito behavioral control, representing an innovative approach that complements traditional strategies.

In this paper, we present an integrated yet stage‐specific discovery framework aimed at contributing alternative strategies for *A. aegypti* control. Importantly, the approaches explored herein do not seek a unified molecular solution or a dual‐target ligand. Instead, they intentionally address two biologically distinct control points: disruption of larval development via interference with JH transport, and modulation of adult blood‐feeding behavior through NPYLR7 agonism. By combining experimentally validated larvicidal screening with computationally driven prioritization of adult‐stage candidates, this work exemplifies a hierarchical pipeline in which different methodological layers contribute complementary insights toward vector control.

## 2 Methods

### 2.1 Virtual Screening of Chalcones by Classical Docking in mJHBP

The three‐dimensional structure of the JH binding protein (mJHBP) of *A. aegypti* was obtained from the Protein Data Bank (PDB) (code 5V13) (Berman 2000), presenting good resolution (1.84 Å). The original crystallographic ligand is JH (JH III). The protein was prepared by removing water molecules and ions, adjusting the pH to 7.5 (*A. aegypti* hemolymph) with APBS software—https://server.poissonboltzmann.org/ (accessed on 15/01/2025), with atomic charges defined by the AutoDockTools‐1.5.7 software (ADT) (Morris et al. 2009). Some visualizations and images were generated by UCFS Chimera‐1.19 software (Pettersen et al. 2004).

The classical virtual screening used 1070 chalcones found in the PubChem database. This chemical class was chosen due to good interactions with mJHBP, previously observed by our research group. The molecules were evaluated at the original JH III binding site, on the 5V13 PDB structure. Molecular docking was performed using AutoDock Vina‐1.5.6 (ADV) (Trott and Olson 2010) and the ADT graphical interface. The grid box was configured to encompass the JH III binding region. To validate the docking method, the original JH III ligand was redocked, and the root mean square deviation (RMSD) was used to measure precision. RMSD values less than 2Å validate the method.

Screening validation was complemented by constructing a Receiver Operating Characteristic (ROC) curve using five active ligands reported as true positives (JH III, Fenoxycarb, Pyriproxyfen, Methoprene, and Hydroprene) and 50 decoys (true negatives) generated on the DUD‐E platform. Model performance was assessed by the Area Under the Curve (AUC).

After these steps, three halogenated chalcones were selected due to promising virtual screening results and their commercial availability (Sigma‐Aldrich): **1c** (4′‐fluoro‐4‐methoxychalcone); **2c** (4’‐chloro‐4‐methoxychalcone); and **3c** (3‐bromo‐4‐methoxychalcone). Visualization of protein‐ligand interactions was aided by BIOVIA Discovery Studio software‐v21.1.0.20298 (BIOVIA 2021).

### 2.2 *In vivo* larvicidal assays

The assays followed an adaptation of the standardized procedure established by the World Health Organization (WHO). Initially, 20 *A. aegypti* larvae (local strain supplied by Moscamed Brasil Biofactory—Juazeiro‐BA, Brazil), at the L2 to L3 stage, were added to each beaker containing 20 mL of mineral water. Each chalcone was initially diluted in DMSO to prepare stock solutions (50 mg/mL). Maintenence was carried out under controlled conditions: temperature of 27°C ± 2°C, relative humidity of 65% ± 10%, and a 12:12 h light:dark photoperiod.

From this, six serial dilutions with distilled water were performed to achieve concentrations in different beakers of 100, 50, 25, 12.5, 6.25, and 3.125 ppm. Each final concentration was obtained in five replicates. The larvicide Spinosad was used as a positive control (PC), and only equivalent DMSO diluted in the beaker water as a negative control (NC), reaching a final DMSO concentration in the beaker of 0.1%. Larval mortality was recorded after 24, 48, and 72 h of exposure as an integrative biological endpoint reflecting severe physiological disruption during larval development. Subsequently, the lethal concentration required to cause 50% lethality (LC₅₀) was calculated.

### 2.3 Deep Learning Based High‐Throughput Virtual Screening in NPYLR7 Receptor

Even with current docking methods optimized to run in seconds on personal computers, virtual screening of large compound libraries still represents a challenge. In this sense, the use of Machine Learning strategies to replace the explicit generation of score functions, as well as the execution of conformational search algorithms, has emerged as an efficient and robust alternative.

In this paper, a 3D model of the NPYLR7 receptor was used to perform a high‐throughput docking screen, aiming to contribute to the identification of potential candidates for inhibiting the *A. aegypti* blood meal. The molecular ligands subjected to screening consisted of just over 1 million compounds obtained from the online ZINC database, filtered by desirable LogP and Molar Weight (MW) values, around values corresponding to a prototype molecule with high affinity for this receptor (Zeledon et al. 2024). The values were LogP up to 3.0 and MW up to 400 Da.

The homology model of *A. aegypti* NPYLR7 was obtained from the GPCR‐I‐Tasser homology modeling server (https://zhanggroup.org/GPCR-I-TASSER/), being this strategy as well as the definition of the connection site for adjusting the grid box necessary for training based on the work of Zeledon et al. (2024).

For the docking of this absurd amount of molecules, we implemented the recurrent neural network (RNN) architecture proposed by Bande and Baday (2024). This Deep Learning methodology, named Attention‐Network, was selected for its proven efficacy in analyzing high‐dimensional and sequential molecular representations. The model integrates a bidirectional Long Short‐Term Memory (LSTM) layer with an adaptive attention mechanism, enabling it to capture complex dependencies and identify chemically significant patterns within the input data.

The molecular featurization, fundamental to the model's performance, was performed using the composite representation strategy from the previous paper (Bande and Baday 2024), which combines three complementary molecular descriptors into a single input vector. This synergistic approach ensures the capture of structural, sequential, and pharmacophoric information. The final input vector, with 4691 features, is the concatenation of the following components:

#### 2.3.1 Morgan Fingerprints

A 1024‐bit binary representation based on substructures, generated from the ECFP algorithm with a radius of 1 (ECFP2). This descriptor is widely recognized for encoding local molecular topologies and structural patterns.

#### 2.3.2 SMILES One‐Hot Encoding

A 3500‐dimensional sequential encoding derived from SMILES strings. Each string is truncated or padded to a fixed length of 100 characters and encoded on a per‐character basis using a vocabulary of 35 chemical symbols. This component preserves the sequential nature of the molecule, which is essential for the LSTM layer.

#### 2.3.3 MACCS Keys

A set of 167 binary structural keys, indicating the presence of sub‐structures relevant in medicinal chemistry. This descriptor provides highly interpretable pharmacophoric information.

The combination of these descriptors results in a robust molecular representation, capable of feeding the neural network with multifaceted data.

The core of the architecture was a bidirectional LSTM layer, configured with 512 hidden units per direction. This layer processes the sequential input vector from both directions, resulting in a hidden state of 1024 dimensions (512 × 2). The bidirectional nature is crucial for capturing long‐range dependencies in sequential representations, such as those derived from SMILES strings.

To ensure a rigorous and unbiased assessment of the model's predictive performance, we adopted a fivefold cross‐validation (K = 5) protocol. This approach is critical for evaluating generalization ability, particularly in regression tasks involving high‐dimensional and chemically diverse molecular datasets. The protocol was executed in five independent iterations. For each fold k∈{1,2,3,4,5}, the model was trained on four of the fivefolds and validated on the remaining fold (k).

The initial docking necessary for RNN training used the Autodock Vina software in a Linux/Ubuntu machine, considering as standard ligands a dataset composed by ten thousand different molecules, added together 30 recognized agonists related by Zeledon and co‐workers (2024), as well as a set of 28 molecules from the Pubchem bioassay database (“Hit Validation Assay to Identify small molecule *A. aegypti* NPYLR7 Agonists ‐ Small Molecule Inhibitors of Mosquito Biting Behavior; BioAssay AID: 1259427, confirmatory) (Wang et al. 2012).

### 2.4 “*De Novo*” Design of Blood Meal Inhibitors (NPYLR7 Agonists)

In this paper, we adopt the method developed in our research group, called DeSAO (“de novo” Drugs using Simulated Annealing (SA) Optimization) (Oliveira Neto et al. 2025), aiming to contribute to new molecular scaffolds that can potentially inhibit the blood meal of *A. aegypti*. This framework stands out for being an efficient and accessible alternative to traditional Deep Learning methodologies, which typically require specialized hardware (such as GPUs) and large volumes of data.

Inspired by the SA algorithm (Kirkpatrick et al. 1983), DeSAO employs a temperature‐based search mechanism to balance the exploration of chemical space. This allows for the generation of optimized molecular candidates using only conventional CPUs, significantly reducing computational costs.

**Figure 1:**
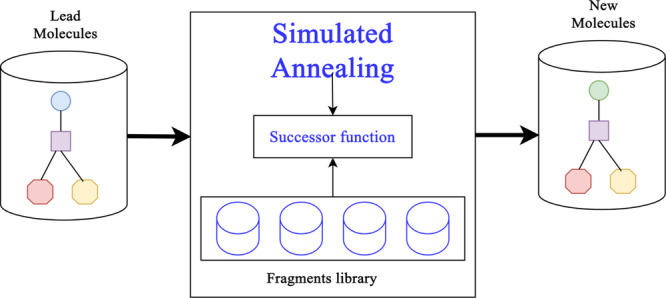
Diagram of the basic architecture of DeSAO. (Adapted from Oliveira Neto et al. 2019).

The DeSAO architecture (Figure 1) begins with two datasets: one of leader molecules, which serves as a starting point, and another used to construct a library of molecular fragments. Each molecule is represented by a binary encoding of structural fragments. This representation facilitates the application of the successor function, responsible for performing controlled modifications (substitutions, additions, or rearrangements) that preserve chemical integrity. This module was reused from the work of Ståhl and co‐workers (Ståhl et al. 2019).

As a leader library for DeSAO, a set of 29 molecules from the paper of Zeledon et al. (2024) was used. This set is analogs designed from the reference compound TDI‐012631, acting as agonists of the NPY‐like receptor 7 (NPYLR7) of *A. aegypti*. All share a quinazoline‐guanidine scaffold, which constitutes the minimum pharmacophore to activate NPYLR7.

A database of 6,000 compounds was employed as a source of molecular fragments, which are molecules optimized for the MCE‐18 descriptor (Molecular Complexity Estimator‐18) (Ivanenkov et al. 2019). This is a cheminformatics metric used to quantify drug‐likeness, capturing key aspects of molecular architecture that differentiate simple, fragment‐like compounds from drug‐like structures with higher pharmacological relevance. Its importance lies in its ability to guide the design of bioactive molecules towards a balance between synthetic feasibility and structural complexity, necessary for selective biological activity.

In DeSAO, the final design was optimized for three properties: Lipophilicity (clogP), Polar Surface Area (PSA), and Molecular Weight (MW). These properties are easily available at RDKit framework, and the desired ranges for each property were: 250 < MW < 500; 0.5 < cLogP < 3.5; 70 < PSA < 180. The chosen ranges provide an overall balance consistent with an optimized pharmacokinetic profile for a bioactive molecule.

The general parameters used in the experiment were: Max Iterations = 4000; Initial Temperature = 30; alpha = 0.9; S = 100; s_temp = 0.5; Sample Size = 512.

## 3 Results

### 3.1 Classical Virtual Screening Results

**Figure 2:**
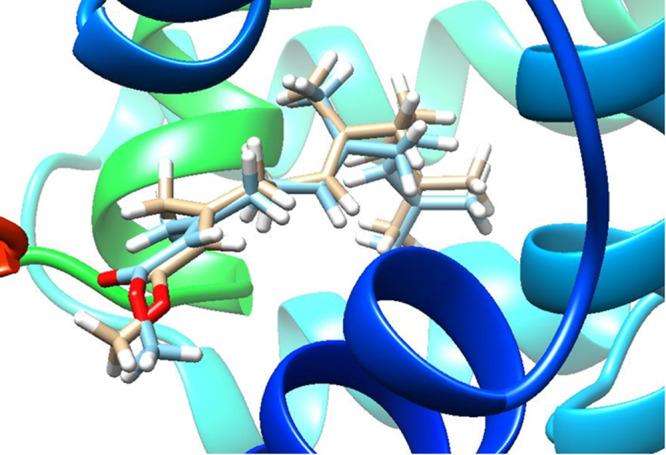
3D structure of crystallographic (beije) and redocked (blue) poses of juvenile hormone binded to mJHBP from *Aedes aegypti*.

**Figure 3:**
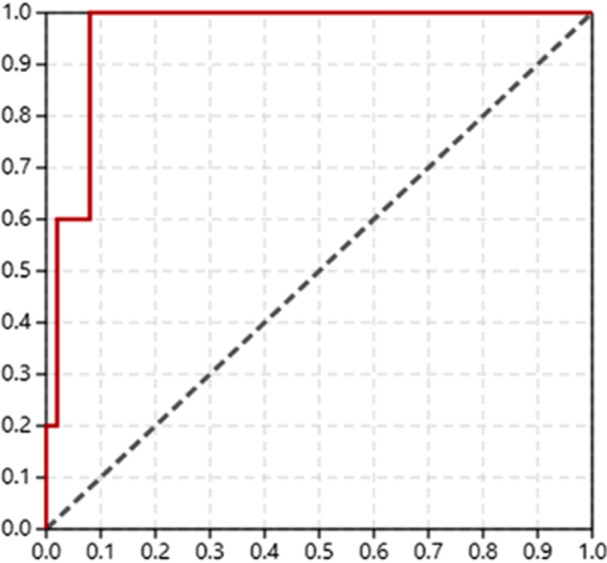
ROC curve obtained from docking pontuations for five true positives and 50 decoys. (y‐axis: fraction of true positives; x‐axis: fraction of false positives (misclassified decoys).

The redocking procedure, involving the crystallographic ligand JH III in the mJHBP protein, resulted in an RMSD = 0.524 Å and binding energy = −8.9 kcal/mol (Figure 2). Being significantly lower than 2 Å, the molecular docking algorithm was considered validated and capable of reproducing the position of the original ligand in the protein binding site. Additionally, the ROC curve constructed from the true positive and decoy ligands resulted in an AUC metric of 0.960. This value falls within the excellent performance range (0.9−1.0), confirming the model's ability to correctly classify true affinities (Figure 3). The energies (kcal/mol) for true ligands were: Fenoxycarb (−9.6); Piryproxyfen (−10.5); Methoprene (−9.3); Hydroprene (−10.5). The juvenile hormone (JH III) was the fifth ligand.

Considering the more than a thousand chalcones used in classic virtual screening calculations, three stood out for having values below −8.9 (JH III ligand score) and being commercially available at affordable prices (Sigma‐Aldrich/Merck). The halogenated chalcones **1c**, **2c**, and **3c** are presented in Table 1.

**Table 1:** Structure and Docking scores for JH III and chalcones selected after Virtual Screening.

Ligand	Binding energy (kcal/mol)
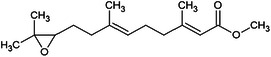 (JH III)Methyl (2E,6E)‐9‐(3,3‐dimethyloxiran‐2‐yl)‐3,7‐dimethylnone‐2,6‐dienoate	−8.9
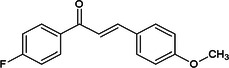 (1c)(E)‐1‐(4‐fluorophenyl)‐3‐(4‐methoxyphenyl)prop‐2‐en‐1‐one	−11.5
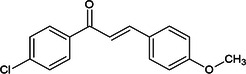 (2c)(E)‐3‐(4‐chlorophenyl)‐1‐(4‐methoxyphenyl)prop‐2‐en‐1‐one	−10.8
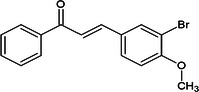 (3c)(E)‐3‐(3‐bromo‐4‐methoxyphenyl)‐1‐phenylprop‐2‐en‐1‐one	−10.0

**Figure 4:**
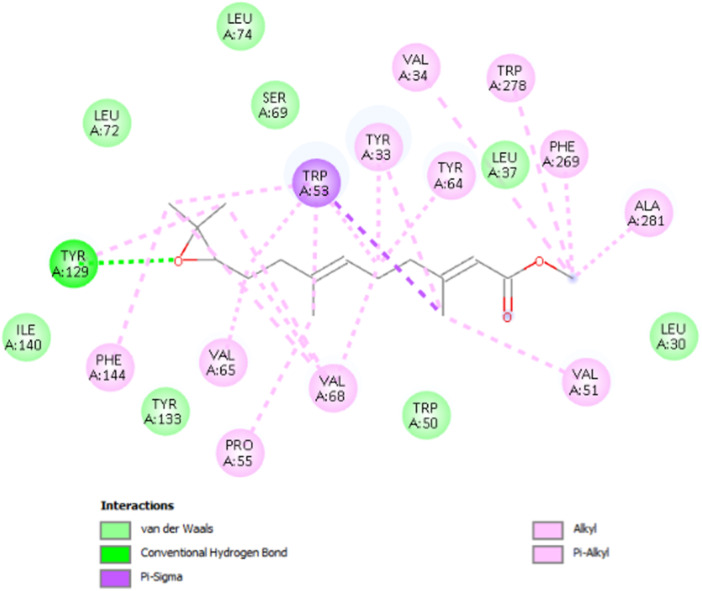
2D interactions map for JH III in the binding site of mJHBP.

The JH binding protein (mJHBP) is found in the hemolymph of the *A. aegypti* mosquito and plays essential roles in the development and reproduction of this mosquito. The mJHBP has only one binding site in its structure, conferring high specificity for the juvenile hormone JH III. Within this protein, JH III performs crucial interactions, being docked in the cavity of the N‐terminal domain, where its epoxy terminus forms hydrogen bonds with residues Tyr‐129. This interaction contributes to the stabilization of the binding between the protein and JH. Furthermore, several hydrophobic residues contribute to the stabilization of the ligand in the protein's binding site, with the main interactions occurring with residues Phe‐144, Tyr‐64, Trp‐53, Val‐65, Val‐68, Leu‐72, Leu‐74, Val‐51, and Tyr‐33 (Figure 4). JH III binding induces a conformational change in mJHBP, resulting in the closure of the binding site, stabilizing the hormone within the protein and making it selective for JH III (Kim et al. 2017).

The relationship between ligand‐protein interactions plays an important role in biological effects and stability within the binding site, including van der Waals (vdW) interactions, hydrogen bonds, and others. These interactions, which occur within the binding site, are not fixed; they can be modulated by the interactions that the protein forms with its ligand. VdW interactions are crucial for stabilization within the binding site, and hydrogen bonds play a crucial role in ligand orientation within the binding site. In the original ligand JH III, a conformational change occurs when it comes into contact with the mJHBP protein, occurring predominantly dispersion interactions and a classical hydrogen bond.

**Figure 5:**
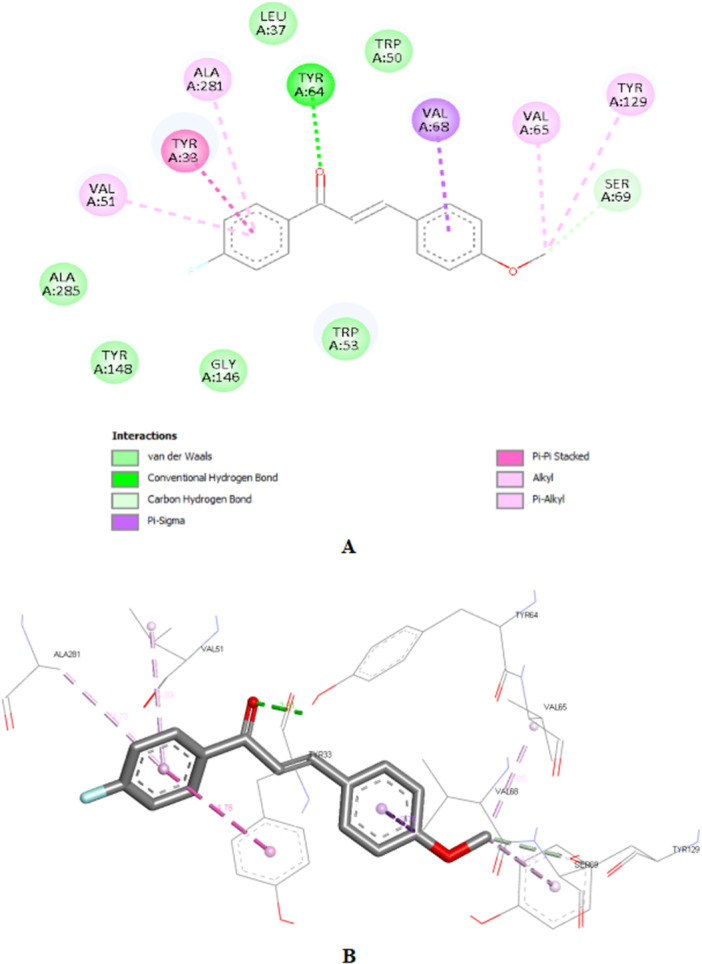
2D interactions map (A) and 3D perspective for 1c in the binding site of mJHBP (B).

Compound **1c**, despite having the best theoretical affinity (−11.5 kcal/mol), showed interactions that differed slightly from JH III. A conventional hydrogen bond with Tyr‐64 (2.30 Å) and a hydrogen‐carbon bond with Ser‐69 were observed. Important hydrophobic interactions included Pi‐Pi stacking with Tyr‐33 and Pi‐alkyl with Val‐51 and Ala‐281. It is interesting to note that the methoxy group and the carbonyl of the chalcone are in the same 3D perspective as the epoxy and carbonyl regions, respectively, of JH III (Figure 5).

**Figure 6:**
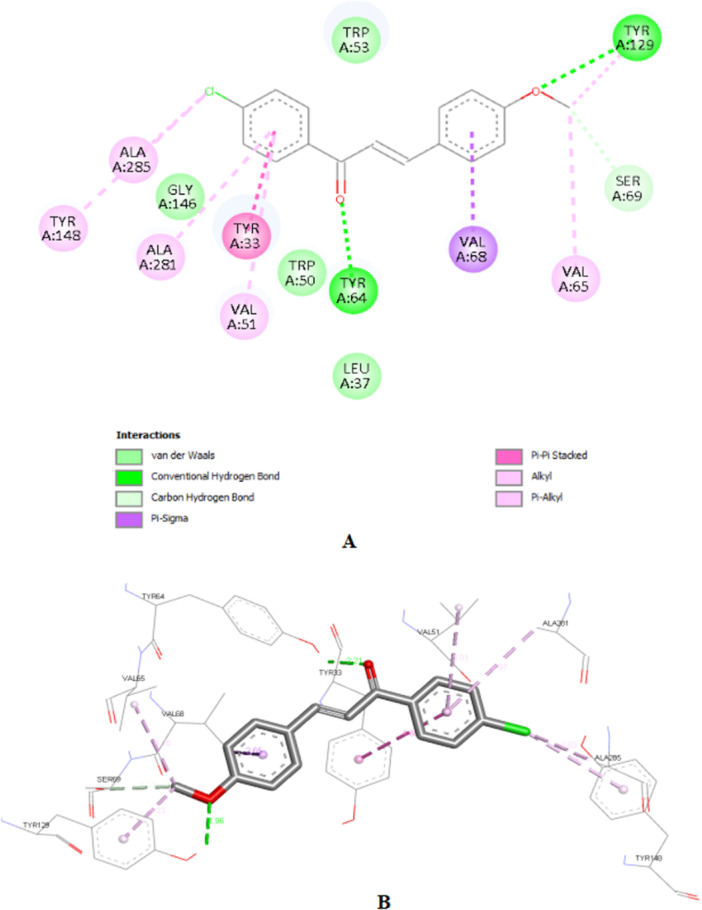
2D interactions map (A) and 3D perspective for 2c in the binding site of mJHBP (B).

Compound **2c** (−10.8 kcal/mol) stood out for maintaining crucial polar interactions. It formed two conventional hydrogen bonds, including one with the key residue Tyr‐129 (2.95 Å) and another with Tyr‐64 (2.21 Å). The interaction with Tyr‐129, which is the same one that stabilizes the epoxide of JH III, confers greater proximity in the binding mode compared to the standard ligand, although in **2c** it occurs with the methoxy group. Hydrophobic interactions also predominate due to its aromatic structure, such as Pi‐Pi stacked with Tyr‐33. The same spatial carbonyl‐methoxy relationship of chalcone is maintained in relation to the carbonyl‐epoxy groups of JH III (Figure 6).

**Figure 7:**
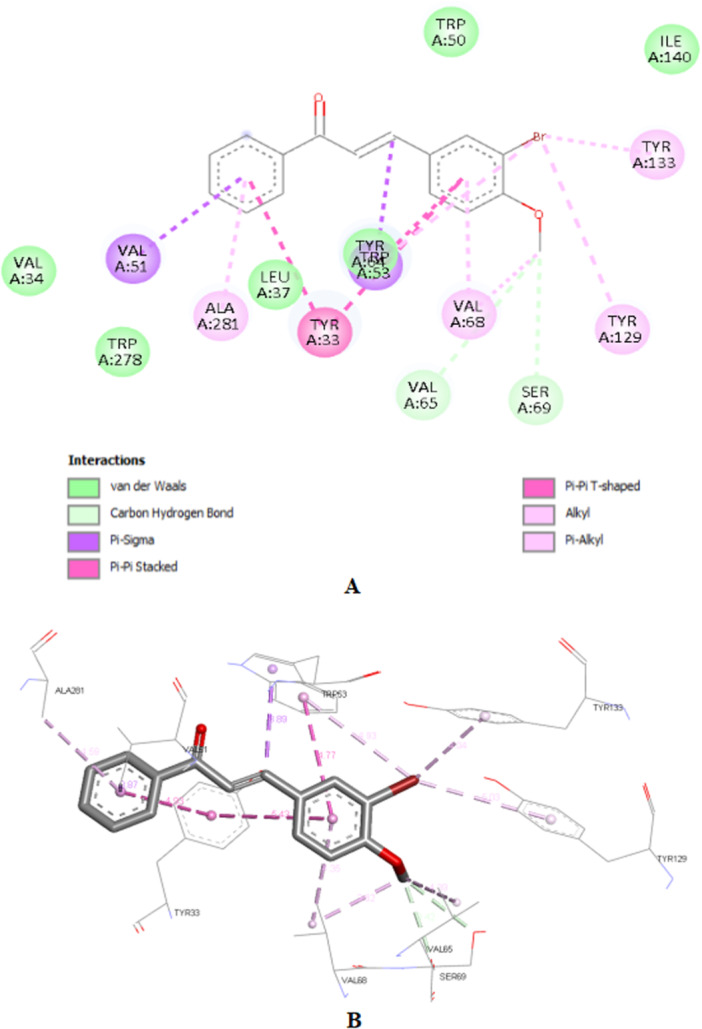
2D interactions map (A) and 3D perspective for 3c in the binding site of mJHBP (B).

Compound **3c** (−10.0 kcal/mol) showed the worst affinity among the halogenated chalcones. In its simulated interactions, no conventional hydrogen bonds were observed, only weak hydrogen‐carbon bonds (Val‐65, Ser‐69). Dispersion interactions such as Pi‐Sigma and Pi‐Pi T‐shaped (Trp‐53) predominated. The presence of the very bulky bromine on the same ring as the methoxy group, unlike other chalcones, appears to impair interactions, in addition to distancing the two anchoring points identified for JH III (carbonyl and epoxy), related to the carbonyl and methoxy, respectively, of each previous chalcone. This results in a more truncated structure than the elongated pattern of **1c** and **2c** with two substituents on different rings. Furthermore, the hydrophobicity that **3c** should exhibit in aqueous solution deserves attention (Figure 7).

### 3.2 In Vivo Larvicidal Effects of Halogenated Chalcones

Larvicidal assays revealed that the effects of halogenated chalcone analogs became evident predominantly after prolonged exposure (72 h) (Table 2). This temporal profile is consistent with a mechanism involving disruption of JH transport and signaling rather than acute toxicity. JH–dependent processes regulate larval growth, molting, and metamorphosis through coordinated transcriptional and physiological cascades, which require time to manifest phenotypically (Noriega and Nouzova 2020). Consequently, interference with mJHBP function is expected to result in cumulative developmental dysregulation, culminating in delayed lethality rather than immediate mortality.

**Table 2:** Larvicidal effects of halogenated chalcones (1c–3c) against *Aedes aegypti* (L2–L3) after 24, 48, and 72 h of exposure.

Concentration (ppm)	Mean and standard deviation			Lethalithy (%)		
	1c					
	24 (h)	48 (h)	72 (h)	24 (h)	48 (h)	72 (h)
100	1 ± 0.7	1.4 ± 0.55	6.6 ± 3.1	5	7	33
50	1 ± 0.7	2 ± 1.58	9.6 ± 3.36	5	10	48
25	0.2 ± 0.45	1 ± 1.41	10 ± 3	1	5	50
12.5	0 ± 0	0.4 ± 0.54	7.2 ± 2.5	0	2	36
6.25	0.2 ± 0.44	0.2 ± 0.44	4.8 ± 0.84	1	1	24
3.125	0.2 ± 0.44	0.6 ± 0.55	0.2 ± 0.45	1	3	1

Concentration (ppm)	Mean and standard deviation			Lethalithy (%)		
	2c					
	24 (h)	48 (h)	72 (h)	24 (h)	48 (h)	72 (h)
100	2 ± 1	3.4 ± 1.51	12.2 ± 1.3	10	17	61
50	1 ± 1.22	5.2 ± 3.9	12.4 ± 3	5	26	62
25	0.2 ± 0.45	2.8 ± 0.84	12.8 ± 2.5	1	14	64
12.5	0.8 ± 0.84	1.6 ± 1.52	12.8 ± 1.3	4	8	64
6.25	0.4 ± 0.55	1.8 ± 1.3	9.6 ± 0.86	2	9	48
3.125	0.4 ± 0.55	1.6 ± 1.14	12 ± 1.58	2	8	60

Concentration (ppm)	Mean and standard deviation			Lethalithy (%)		
	3c					
	24 (h)	48 (h)	72 (h)	24 (h)	48 (h)	72 (h)
100	1 ± 1.41	1.2 ± 1.78	1.4 ± 1.67	5	6	7
50	0 ± 0	0 ± 0	0.8 ± 0.44	0	0	4
25	0.4 ± 0.55	0.4 ± 0.55	0.4 ± 0.55	2	2	2
12.5	0 ± 0	0 ± 0	0.4 ± 0.55	0	0	2
6.25	0 ± 0	0.2 ± 0.45	0.2 ± 0.45	0	1	1
3.125	0 ± 0	0 ± 0	0.2 ± 0.45	0	0	1

This delayed larvicidal action aligns with extensive literature demonstrating that perturbation of JH signaling produces systemic developmental defects rather than immediate toxicity (Jindra et al. 2013). Studies have shown that interference with JH transport or receptor‐mediated signaling can result in impaired molting, abnormal larva to pupa transitions, growth arrest, and eventual lethality. Thus, the mortality observed after 72 h likely reflects the cumulative physiological consequences of disrupted hormonal homeostasis, consistent with the biological role of mJHBP in insect development.

After a logarithmic regression (Finney's table ‐ probit), the LC_50_ for **1c** was estimated at 34.06. However, the LC_50_ for **2c** and **3c** was not determined because the effects at all concentrations were pratically all above 50% for one and very low for the last, with mortalities below 50%. For **1c**, we found that the low mortality at the highest concentration (100 ppm), followed by an increase at 50 ppm, and the subsequent expected decrease, was due to limited solubility initially at 100 ppm. Therefore, the first value was not considered in the logarithmic regression. For **2c**, all mortality percentages remained within the 60% range, even at lower concentrations. Thus, even though its LC_50_ value has not been obtained, we can attest to its great superiority in relation to other analogues.

The results demonstrate that, although the fluorinated chalcone (**1c**) exhibited the best affinity in silico, the chlorinated chalcone (**2c**) with nearby energy demonstrated greater efficacy in vivo (Table 2). This observation highlights widely recognized limitations of docking‐based affinity estimates, which do not fully incorporate factors such as compound solubility, lipophilicity‐modulated bioavailability, metabolic stability, and the dynamic nature of protein‐ligand interactions in biological systems. While docking scores are useful tools for ranking and prioritizing candidates, they represent a simplified energetic approximation and should not be interpreted as strict direct predictors of biological activity. In this context, the superior performance of compound 2c in in vivo assays may reflect a more favorable balance between molecular recognition and physicochemical properties governing overall behavior in the larval system. Thus, the slight divergence between computational predictions and experimental results confirms the importance of investigating multiple analogues when confirming trends predicted in silico.

The success of compound **2c** may be related to its ability to form a conventional hydrogen bond with the Tyr‐129 residue, the same amino acid used by the original ligand JH III. On the other hand, the poor larvicidal performance of compound **3c** may be attributed to its low water solubility and, according to the in silico data, the absence of a conventional hydrogen bond at the binding site, which is crucial for the ligand's stability and orientation. The integration of computational tools and biological analyses reinforces the potential of chlorinated chalcone analogs as larvicidal agent, suggesting the involvement of the JH transport pathway.

Although the experimental evaluation focused on larval mortality as the primary outcome, it is important to consider this result in light of the biological role of JH‐binding protein (mJHBP). This protein is essential for the transport, stabilization, and regulated release of JH, which orchestrates key processes in insect growth, molting, and metamorphosis. Therefore, interference with mJHBP function is expected to result in systemic developmental dysregulation, rather than a single isolated physiological effect. Previous studies have demonstrated that disruption of JH signaling pathways can lead to delayed or incomplete molting, abnormal larva‐to‐pupa transitions, growth arrest, and developmental lethality. In this context, the mortality observed in the present study likely represents the end result of cumulative physiological and developmental disturbances induced by impaired hormone transport and signaling, considering the lethality effectiveness only after 72 h.

We acknowledge that the present study did not include direct monitoring of sublethal developmental phenotypes, such as molting abnormalities, growth retardation, or morphological defects. Future studies incorporating histological analyses, developmental staging, and measurements of hormone levels will be important to better delineate the mechanistic consequences of mJHBP modulation at the organism level.

### 3.3 High Throughput Virtual Screening (HTVS) Results

**Figure 8:**
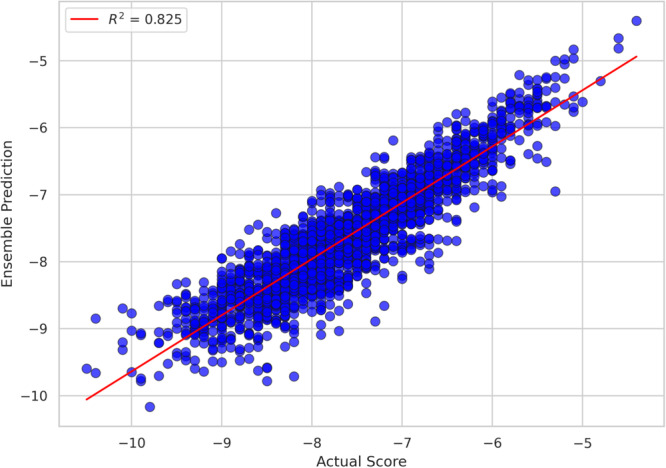
Scatter plot of standard docking score values (actual energies obtained with Vina) versus those predicted by the RNN‐LSTM architecture.

The trained RNN‐LSTM model showed consistency in performance metrics across different validation folds (Table 3). For the best visualization, Figure 8 consolidates the results from all folds into a single scatter plot, where the proximity of the points to the regression line reflects the accuracy of the predictions.

**Table 3:** Validation metrics for RNN model.

Fold	MSE	MAE	*R*²
1	0.188	0.343	0.788
2	0.199	0.347	0.784
3	0.189	0.334	0.779
4	0.176	0.327	0.808
5	0.243	0.384	0.740

Abbreviations: MAE = Mean Absolute Error, MSE = Mean Squared Error, *R*² = Coefficient of determination.

It is important to emphasize that a virtual screening of 1 million small molecules using only the original Autodock Vina algorithm and scoring functions would take approximately 115 days, considering an average individual docking calculation time of 10 s on personal computers with CPUs. We must also consider that the machine would have to be running for the entire duration. In this sense, the application of the RNN‐LSTM architecture in this work allowed the scanning of a gigantic sample space in a few minutes, using a workstation with 32 GB of RAM and an NVIDIA RTX 4070 Ti GPU (16 GB).

The spreadsheet of results after RNN‐LSTM showed 124 molecules (out of more than 1 million) with binding energies (scores) below −9.9 kcal/mol, thus being considered the best assertions. A Python code using RDKit allowed comparison between this set of molecules generated after HTVS and two standard databases, one corresponding to the 30 NPYLR7 agonists from the paper by Zeledon et al. (2024) and another of 28 molecules from a confirmatory NPYLR7 affinity bioassay, from the PubChem database (PubChem AID: 1259427). Similarity coefficients were calculated using three distinct types of molecular fingerprints available in RDKit: Morgan, RDK, and MACCS. For each pair of molecules, fingerprints were generated individually and then compared using the Tanimoto metric, allowing a quantitative assessment of the structural similarity between the compounds, with values of 0.224, 0.332, and 0.574, respectively for the best match, which occurred with one of the compounds from the set of 28. This approach provides a complementary analysis, since each type of fingerprint captures different aspects of the chemical structure.

**Figure 9:**
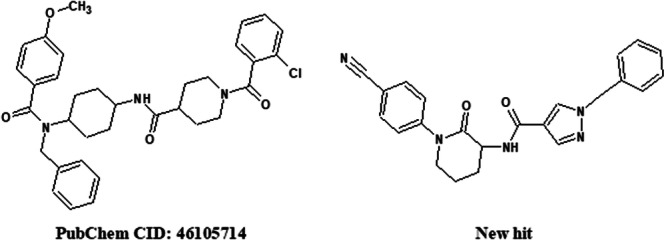
Simplified 2D structural diagram of the new compound identified after HTVS (new hit) and the structurally related compound from the set of 28 known bioactive compounds (PubChem CID: 46105714).

Figure 9 allows for a visual comparison between the two structures with the best correspondence, revealing aspects relevant to understanding the potential biological activity of the new hit against the NPYLR7 receptor, associated with the feeding behavior of *A. aegypti*. Presenting a more compact architecture, the new hit (from HTVS) maintains key structural elements that may be associated with possible receptor recognition, such as the presence of a central amide function between two rings, where one of them is nitrogen‐containing in both compounds, in addition to aromatic systems at both ends of both molecules. At one of these ends, the standard bioactive compound has an amide carbonyl group, and the hit has a nitrile function, which may perform similar functions from a stereoelectronic and molecular recognition point of view. Finally, the lower structural complexity of the hit identified via RNN‐LSTM represents advantages in terms of cost and time in synthetic production, corresponding to an interesting simplification compared to a compound with a recognized agonist effect on NPYLR7. Thus, future experimental studies may confirm or refute this hypothesis, potentially opening avenues for alternatives that inhibit *A. aegypti* feeding.

### 3.4 De Novo Design Results

The set of 29 molecules from the paper of Zeledon et al. (2024) were used as a library of leads, acting as agonists of the NPYLR7 of *A. aegypti*. All share a quinazoline‐guanidine scaffold, which constitutes the minimum pharmacophore of this dataset to activate NPYLR7. The compounds had EC₅₀ values from 39.3 µM to 1.92 nM, in addition to an NC with EC₅₀ > 100 µM, without the guanidine moiety. These molecules were tested in a miniport olfactometer (host‐seeking for CO₂ + human odor), and then a subset was evaluated in a “mouse‐in‐cage” assay (to see if they actually blocked blood feeding). Despite the presence of very potent molecules in vitro (up to EC₅₀ = 1.92 nM), only 3 out of the 30 consistently reduced blood feeding in vivo when administered at 1 µM: TDI‐014184, TDI‐014186, and TDI‐014188.

The three molecules above have some fine adjustments in their structure, such as the presence of a spacer linked to tetrahydropyran, phenyl, or a tetrahydroisoquinoline system, all at carbon 7 of the quinazoline ring. However, it should be noted that there was no direct linear correlation between higher potency in vitro and in vivo results. It can be said that TDI‐014188 stands out as the best compromise between higher potency in vitro (60.2 nM) and strong suppression of stinging and blood‐feeding behavior at 1 µM.

**Figure 10:**
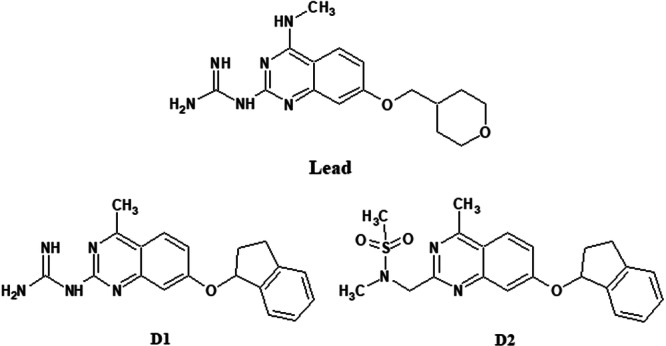
The most active molecule in vivo from the work of Zeledon et al. (2024) (“Lead”) and two new architectures generated by DeSAO (D1 and D2). (Compounds D1 and D2 are highlighted as representative examples due to their increased structural novelty while preserving key pharmacophoric features of the lead series).

After applying DeSAO, the code managed to generate 22 analogs containing structural relationships with the leader set and merging desirable fragments in this optimization. These analogs represent a pre‐filtered chemical set that simultaneously satisfies predefined physicochemical windows (MW, cLogP, and PSA) and fragment‐based complexity constraints (MCE‐18). Rather than serving as a final ranking, this set is intended to provide chemically feasible and biologically plausible starting points for subsequent prioritization and experimental evaluation. Among these molecules, the appearance of the dihydro‐indene pattern on the opposite side to guanidine stands out, as well as the appearance of the sulfamate function replacing guanidine, representing innovative characteristics that can be explored in future in vitro/in vivo assays. These more differentiated hits are illustrated in Figure 9, and the SMILES with the other compounds most similar to the leader dataset can be viewed in the supporting material (Figure 10).

It is important to emphasize that both NPYLR7‐oriented HTVS and DeSAO pipelines were conceived as hypothesis‐generating and candidate‐prioritization tools, rather than as a direct demonstration of biological efficacy. The computational models were trained and benchmarked using experimentally validated agonists reported in the literature and in PubChem BioAssay, ensuring biological relevance at the data level. However, functional validation of the newly proposed candidates, such as receptor activation assays and behavioral blood‐feeding inhibition tests, remains outside the scope of the present study and will be addressed in future work.

## 4 Conclusion

This study demonstrates the value of integrating computational chemistry, machine learning, and in vivo bioassays within a hierarchical and stage‐specific discovery framework for *A. aegypti* control. While the chalcone/mJHBP axis establishes a complete computational–experimental link through validated larvicidal assays, the NPYLR7‐oriented Deep Learning and DeSAO pipelines provide biologically grounded candidate prioritization for adult‐stage intervention. Together, these complementary strategies illustrate how differing levels of experimental maturity can be coherently integrated to guide future vector control research.

Classical docking approaches identified halogenated chalcones with strong predicted affinity for the mJHBP protein, and subsequent larvicidal assays confirmed the biological relevance of some of these predictions. Notably, 4′‐chloro‐4‐methoxychalcone (**2c**) maintained consistent lethality across concentrations, aligning with its ability to reproduce key interactions essential for JH III recognition. These findings reinforce the JH transport pathway as a susceptible larval target and highlight chalcones as promising scaffolds for further optimization.

At the adult stage, the application of Deep Learning played a central role in expanding the chemical search space and accelerating candidate prioritization beyond the limits of conventional docking‐based screening. Importantly, the HTVS strategy employed herein was designed as a hypothesis‐generating tool trained on experimentally validated agonists, enabling rapid identification of chemically plausible NPYLR7‐oriented candidates rather than direct prediction of biological efficacy. The RNN‐LSTM architecture with an attention mechanism enabled the prediction of docking scores for more than one million compounds within minutes, a task that would require over one hundred days of CPU‐based calculations using AutoDock Vina. This high‐throughput, AI‐accelerated screening produced a chemically diverse subset of prioritized candidates with potential NPYLR7 agonistic activity, demonstrating the effectiveness of neural sequence models in learning stereoelectronic and structural features relevant to GPCR binding.

Complementarily, DeSAO successfully generated novel molecular architectures that maintain essential pharmacophoric elements of other known NPYLR7 agonists while introducing meaningful structural innovations, such as the emergence of dihydroindene‐based and sulfamate‐containing motifs. These findings underscore the value of combining classical heuristic optimization and modern neural network–based prediction within the same workflow.

Together, these results provide a multi‐layered framework connecting molecular design, docking approaches, and biological validation. Integration is essential for developing next‐generation control agents that are mechanistically informed, selective, and capable of complementing existing vector‐management strategies. Future work can focus on expanding structure–activity relationships, validating NPYLR7 hits in functional assays, and further optimizing chalcone analogues for potency, stability, and environmental compatibility.

Although the integrated strategy presented herein spans multiple biological targets and methodological levels, it is important to acknowledge differences in experimental maturity between the investigated axes. The mJHBP/chalcone component establishes a complete computational/biological link through *in vivo* larvicidal assays, whereas the NPYLR7 focused deep learning and DeSAO workflows provide a biologically grounded prioritization of candidate molecules. Definitive confirmation of adult‐stage efficacy will require subsequent functional assays, including NPYLR7 activation and blood‐feeding inhibition experiments.

### 4.1 Use of Artificial Intelligence Tools

Some parts of the manuscript in Introduction and Discussion sections were written with the assistance of ChatGPT (OpenAI, San Francisco, CA, USA, GPT‐4, May 2025), to improve clarity and grammar. Similarly, some scripts for algorithms were written with the assistance of ChatGPT. All content was reviewed and revised by the authors to ensure accuracy and originality.

## Author Contributions

**Herbert Bezerra Leite:** investigation, methodology. **Filipe Alves Ribeiro Rodrigues:** investigation, methodology. **Luana Beatriz Rocha Silva:** investigation, methodology. **Vanessa Costa Santos:** investigation, methodology. **Rosalvo F. Oliveira Neto:** conceptualization, writing – original draft, project administration, supervision, formal analysis. **Edilson B. Alencar Filho:** conceptualization, writing – original draft, funding acquisition, project administration, supervision, formal analysis.

## Conflicts of Interest

The authors declare no conflicts of interest.

## Supporting information

supplementary_material_herbert.
